# Risk factors for mild cognitive impairment in patients with age-related hearing loss: a meta-analysis

**DOI:** 10.1016/j.bjorl.2024.101467

**Published:** 2024-07-11

**Authors:** Chenxingzi Wu, Wenjuan Wang, Ruilin Li, Yuhong Su, Huiling Lv, Shuhong Qin, Zhanhang Zheng

**Affiliations:** Guangxi University of Chinese Medicine, Nanning, Guangxi, China

**Keywords:** Age-related hearing loss, Mild cognitive impairment, Risk factors, Meta-analysis

## Abstract

**Objectives:**

One of the most common sensory impairments in the elderly is age-related hearing loss, and individuals with this condition have a higher risk of mild cognitive impairment than the overall aged population. The purpose of this study was to conduct a systematic review of the literature in order to evaluate the evidence supporting the hypothesis that mild cognitive impairment may be developed in patients with age-related hearing loss.

**Methods:**

The PRISMA principles were followed when searching the databases of the China Knowledge Network, Wanfang, China Biomedical Literature Database, Pub Med, Cochrane Library, Embase, and Web of Science. Two investigators independently carried out the quality assessment, data extraction, and literature review of the eligible studies. Stata 17.0 was used to finish the statistical analysis and descriptive results.

**Results:**

A total of 13 articles containing 2,222,036 individuals who were evaluated for demographic traits, factors associated with age-related hearing loss, vascular neurologic factors, and psychological factors were included after 2166 search records were found in the database. In patients with age-related hearing loss, eleven factors were found to be risk factors for the development of mild cognitive impairment: age (OR = 1.63; 95% CI 1.09–2.43), male (OR = 1.29; 95% CI 1.14–1.47), degree of hearing loss (OR = 1.35; 95% CI 1.03–1.75), not wearing hearing aids (OR = 1.56; 95% CI 1.37–1.79), cerebrovascular disease (OR = 1.41; 95% CI 1.17–1.69), cardiovascular disease (OR = 1.29; 95% CI 1.07–1.55), diabetes mellitus (OR = 1.28; 95% CI 1.20–1.35), head injury (OR = 1.22; 95% CI 1.13–1.33), alcohol consumption (OR = 1.28; 95% CI 1.14–1.43), and tobacco use (OR = 1.19; 95% CI 1.14–1.25), and depression (OR = 1.63; 95% CI 1.47–1.81).

**Conclusion:**

Caregivers can customize care strategies to decrease the occurrence of mild cognitive impairment in elderly deaf patients by considering demographic traits, factors associated with age-related hearing loss, vascular-neurologic factors, and psychological factors.

## Highlights

• Patients with age-related hearing loss are prone to mild cognitive impairment.
• Age, depression are significant factors in hearing loss patients’ cognition.
• Demographic, hearing, vascular-neurologic, and psychological factors deserve attention.

## Introduction

Age-related hearing loss, also known as ARHL, is a common sensory condition that affects over one-third of those aged 65 and older worldwide.^1^ ARHL results in sensory decline, reduced social interaction, and unpleasant emotional states such as loneliness and sadness.^1^, ^2^ These effects not only diminish the overall well-being of individuals, but have also been established as an autonomous risk factor for cognitive decline.^3^ However, Mild Cognitive Impairment (MCI) is a condition characterized by a deterioration in cognitive abilities that falls between normal aging and dementia.^4^ It is considered an early stage of dementia and its occurrence becomes more common as individuals age. The prevalence of MCI among those aged 60 years or older ranges from 6.7% to 25.2%.^5^, ^6^, ^7^ MCI results in a decline in self-care abilities, places a significant strain on family caregivers, and incurs substantial financial costs, estimated to be around $1 trillion annually worldwide.^3^ Therefore, it is crucial to identify the risk factors for MCI in patients with ARHL to benefit both patients and their families.

While there is a significant body of literature that explores the risk factors for ARHL and MCI separately, there are few comprehensive studies that investigate the connection between these two conditions and how ARHL may contribute as a potential risk factor for MCI. Additionally, there is inconsistency in the literature regarding the reported risk factors for the development of MCI in individuals with ARHL, and the predictive value of certain factors remains controversial.

The aim of this study is to evaluate the evidence supporting ARHL as a possible risk factor for the onset of MCI by conducting a comprehensive assessment of the available literature. In order to ascertain the correlation between an elevated risk of MCI and varying degrees of hearing loss, we will analyze and compare multiple studies to uncover commonalities and disparities. Furthermore, we will assess potential factors that may moderate the association between ARHL and MCI, including demographic traits, factors associated with ARHL, vascular neurologic factors, and psychological factors.

## Methods

The protocol for this systematic review is filed in the PROSPERO database, specifically in the International Registry of Prospective Systematic Reviews, under the code CRD 42024507001.

### Search strategy

The investigation was carried out in accordance with the principles provided by the Preferred Reporting Items for Systematic Reviews and Meta-Analyses (PRISMA).^8^ A comprehensive search was conducted to find literature on risk factors for MCI in patients with ARHL. The search included databases such as China Knowledge Network, Wanfang database, China Biomedical Literature Database, Pub Med, Cochrane Library, Embase, CINAHL, and Web of Science. Additionally, references cited in the included literature were also searched retrospectively. The search time spanned from the inception of the database to October 26, 2023. The search method was established by combining subject terms with free terms and tailored to suit the unique attributes of each database. The precise search approach is displayed in attachment 1.

### Inclusion and exclusion criteria

The inclusion criteria for this study were as follows: (1) the study design was either a cohort study or a case-control study; (2) the study participants were diagnosed as senile deaf patients based on the diagnostic criteria for hearing loss outlined in the World Hearing Report published by the World Health Organization in 2021^9^; (3) the outcome measure was MCI, diagnosed according to the diagnostic criteria provided by the U.S. National Institute on Aging and Alzheimer’s Disease Association (NIA-AA), the Chinese Alzheimer’s Disease Association, and Petersen’s criteria.^10^, ^11^, ^12^ Exclusion criteria: (1) literature written in languages other than Chinese and English; (2) repetitive publications and conference papers; (3) unable to access the entire text; (4) insufficient raw data and unable to extract data.

### Study selection and data extraction

Two scholars conducted a thorough examination of the literature using specific criteria. After removing duplicates with the help of endnote 21, the initial screening was conducted by reviewing the title and abstract to exclude literature that did not align with the study’s topic, study type, or study population. The entire content was thoroughly read and reviewed again. When there is a disagreement, consult with the third researcher. The two researchers reviewed and extracted relevant information from the literature, including: (1) fundamental details of the included studies such as authors, year of publication, study location, study type, and sample size; (2) baseline characteristics of the study population; (3) important aspects of the risk of bias assessment; and (4) Odds Ratios (OR) with 95% Confidence Intervals (95% CI) for each influencing factor.

### Quality assessment

The Newcastle–Ottawa Scale (NOS) is a technique recommended by the Cochrane Collaboration for assessing the risk of bias in observational studies.^13^ Two researchers used the NOS to evaluate the quality of the literature.

### Statistical analysis

A meta-analysis was conducted using Stata 17 to analyze the retrieved data. The OR was utilized as the statistical measure of effect analysis, and it 95% CI was reported. The I^2^ statistic was used to analyze heterogeneity in the literature that met the inclusion criteria. An I^2^ value below 50% indicates that there is no variation across the studies included in the analysis. On the other hand, an I^2^ value above 50% suggests that there is statistical variation among the studies. In order to identify the cause of this variation, sensitivity analyses were conducted. If the major clinical heterogeneity was accounted for, and there was still a substantial heterogeneity in the data, a random-effects model was employed. On the other hand, a fixed-effects model was employed. The study included individual risk factor analysis literature with fewer than 10 studies for the meta effect size combination. However, no publication bias funnel plot analysis was conducted. Statistical significance was determined for differences at a *p*-value of less than 0.05.

## Results

### Study selection

**Figure 1:**
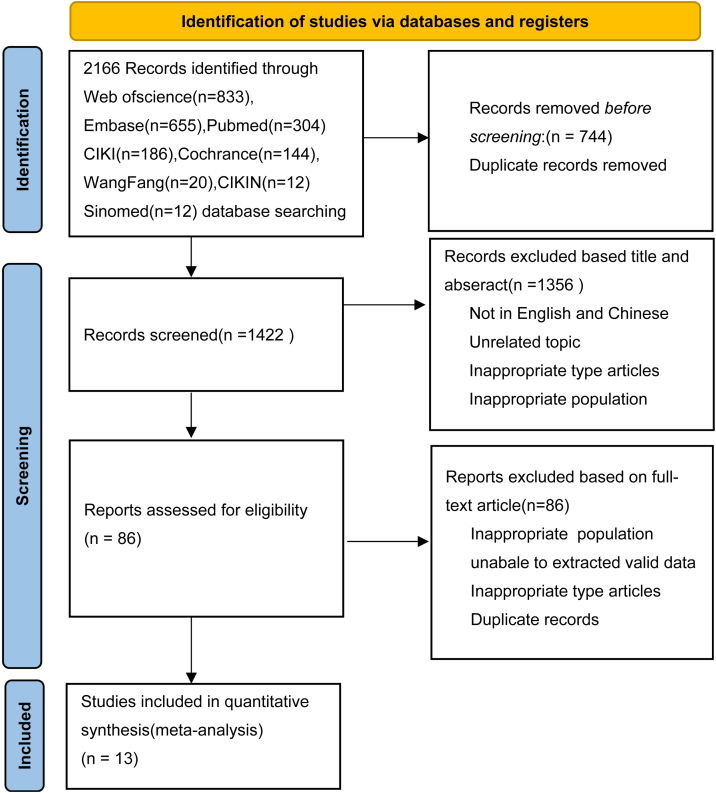
Flow chart for literature screening.

The search data produced a grand total of 2166 documents, with 744 duplicates being removed. After reviewing the titles and abstracts, a total of 86 papers were found to meet the criteria for full text screening. Ultimately, only 13 of these documents were deemed suitable for inclusion. Fig. 1 displays the PRISMA flowchart.

### Basic characteristics of the included literature and quality evaluation results

A total of thirteen literary works were incorporated, spanning from 2013 to 2023, with a sample size ranging from 200 to 2,060,801 cases. These included three case-control studies and ten cohort studies, consisting of two Chinese literary works and eleven English literary works. Table 1 displays the fundamental attributes of the literature that is covered. The quality evaluation results varied between 5 and 8, with all of them being studies of medium to high quality. The findings of the quality evaluation for the included literature can be found in Table 2.

**Table 1:** Basic characteristics of the included studies.

Author	Year	Country	Sample size	Sambling method	Risk factor
Lin et al.^14^	2013	America	1984	Cohort study	2
Su et al.^15^	2017	China	8121	Cohort study	1, 4, 5, 6, 7, 8, 11
Liu et al.^16^	2019	China	16,270	Cohort study	1, 3, 4, 6, 7, 8, 9
Gao et al.^17^	2020	China	14,309	Cohort study	5
Brewster et al.^18^	2021	America	8529	Cohort study	1, 3
Chen^19^	2021	China	10,341	Cohort study	3
Dai et al.^20^	2021	China	200	Case-control study	1, 2, 10, 12
Byun et al.^21^	2022	Korea	17,560	Cohort study	1, 3, 4, 5, 6, 7, 9
Chern et al.^22^	2022	America	2,060,801	Cohort study	1, 4, 8, 11.
Stevenson et al.^23^	2022	England	82,039	Cohort study	2, 3, 10
Zhang et al.^24^	2022	Japanese	1453	Case-control study	2
Ganbo et al.^25^	2023	China	429	Cohort study	1
Pan et al.^26^	2023	China	224	Case-control study	2, 12

(1) Age; (2) degree of hearing loss; (3) depression; (4) cerebrovascular disease; (5) alcohol consumption; (6) head injury; (7) diabetes mellitus; (8) cardiovascular disease; (9) male; (10) not wear hearing aids; (11) tobacco use; (12) course of hearing loss.

**Table 2:** Quality evaluation results.

Author	Year	Selection	Comparability	Outcome	Total
Lin et al.^14^	2013	4	2	2	8
Su et al.^15^	2017	4	1	3	8
Liu et al.^16^	2019	3	2	3	8
Brewster et al.^18^	2021	4	1	3	8
Gao et al.^17^	2020	3	1	3	7
Chen^19^	2021	4	2	2	8
Dai et al.^20^	2021	3	1	3	7
Byun et al.^21^	2022	3	1	3	7
Chern et al.^22^	2022	4	2	2	8
Stevenson et al.^23^	2022	4	1	3	8
Ganbo et al.^25^	2023	3	0	2	5
Zhang et al.^24^	2022	3	1	2	7
Pan et al.^26^	2023	3	2	2	7

## Meta-analysis results

### Demographic characteristics

**Figure 2:**
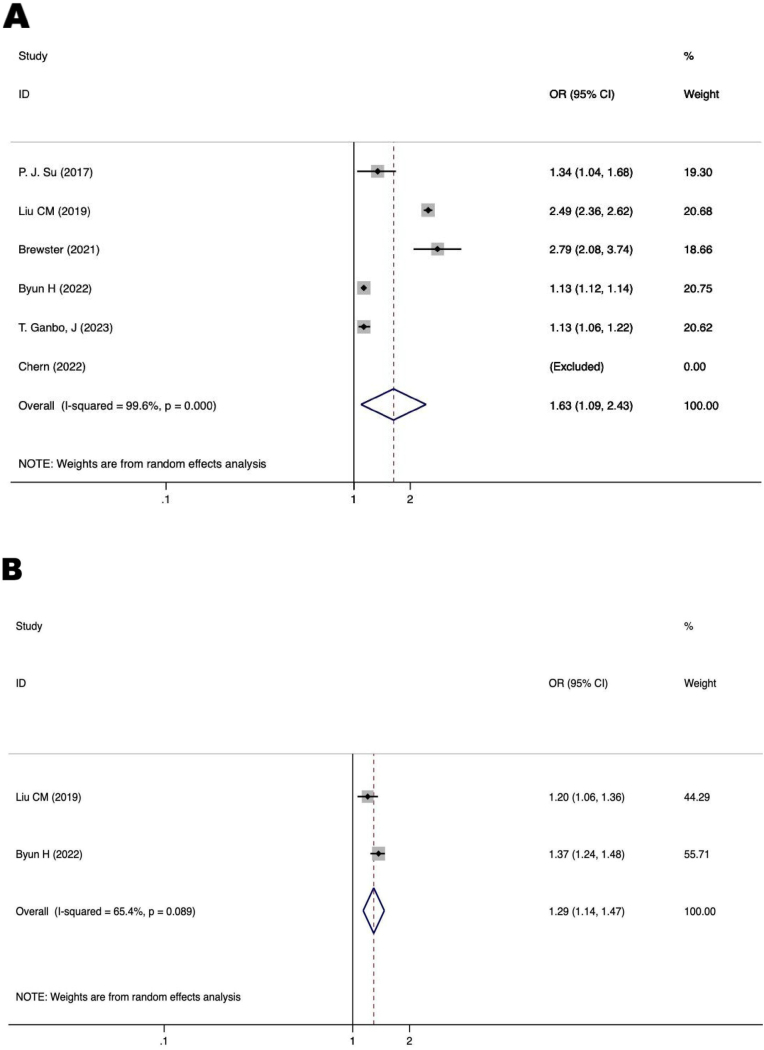
Forest plot of the relationship between demographic traits and MCI in patients with ARHL. (A) Relationship between age and MCI in ARHL patients. (B) Relationship between males and MCI in patients with ARHL. OR, Odds Ratios; CI, Confidence Interval.

Fig. 2 displays a forest plot illustrating the correlation between two parameters associated to demographic and the occurrence of MCI in individuals with ARHL. The risk variables for MCI in individuals with ARHL were shown to be the age (OR = 1.63, 95% CI 1.09–2.43, *p* = 0.017) and male (OR = 1.29, 95% CI 1.14–1.47, *p* < 0.00001).

### Factors associated with age-related hearing loss

**Figure 3:**
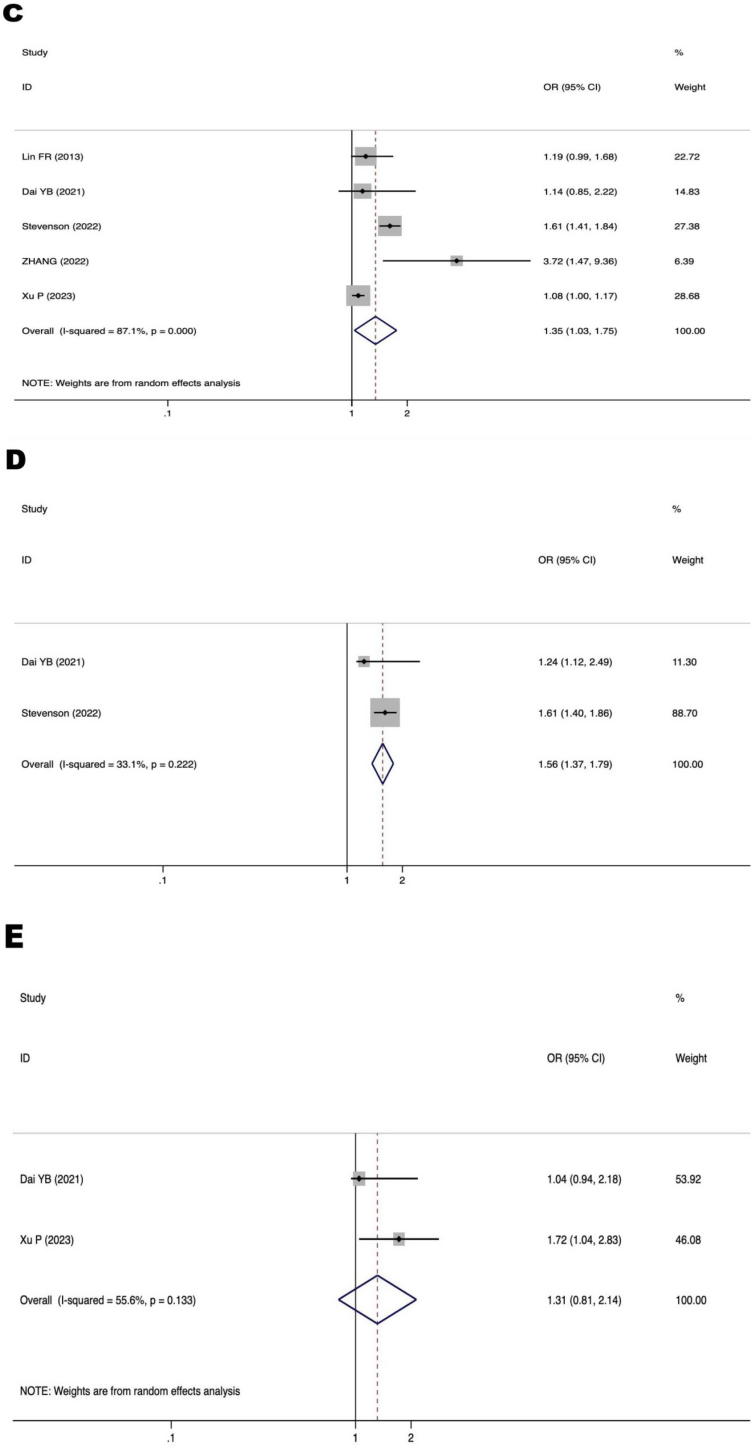
Forest plot of the relationship between ARHL-related factors and MCI in patients with ARHL. (C) Relationship between degree of hearing loss and MCI in patients with ARHL. (D) Relationship between not wearing hearing aids and MCI in patients with ARHL. (E) Relationship between the course of hearing loss and MCI in patients with ARHL. OR, Odds Ratios; CI, Confidence Interval.

Fig. 3 displays a forest plot illustrating the correlation between three parameters associated to ARHL and the occurrence of MCI in individuals with ARHL. The risk variables for MCI in individuals with ARHL were shown to be the degree of hearing loss (OR = 1.35, 95% CI 1.03–1.75, *p* = 0.028) and not using hearing aids (OR = 1.56, 95% CI 1.37–1.79, *p* < 0.00001). Further investigation is required to explore the relationship between prolonged course of hearing loss (OR = 1.31, 95% CI 0.81–2.14, *p* = 0.271) and MCI in patients with ARHL.

### Vascular neurological factors

**Figure 4:**
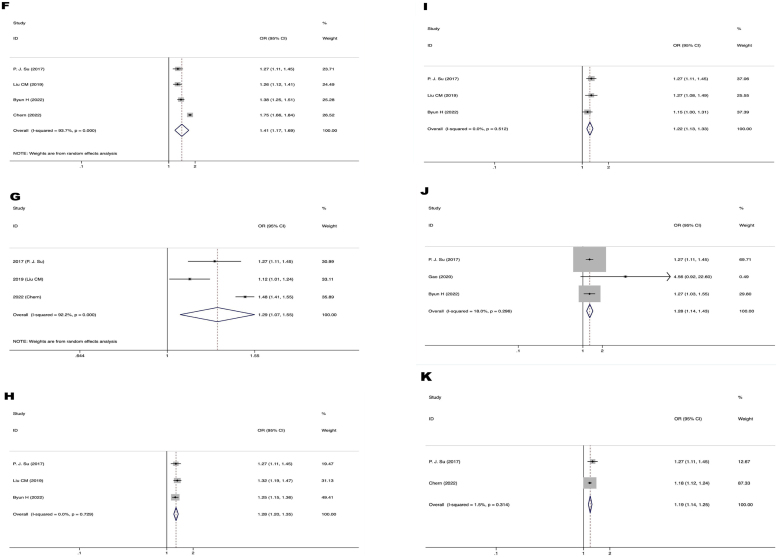
Forest plot of the relationship between vascular neural factors and MCI in patients with ARHL. (F) Relationship between cerebrovascular disease and MCI in patients with ARHL. (G) Relationship between cardiovascular disease and MCI in patients with ARHL. (H) Relationship between diabetes mellitus in relation to MCI in patients with ARHL. (I) Relationship between head injury and MCI in patients with ARHL. (J) Relationship between alcohol consumption and MCI in patientswith ARHL. (K) Relationship between tobacco use and MCI in patients with ARHL. OR, Odds Ratios; CI, Confidence Interval.

Fig. 4 displays a forest plot illustrating the correlation between six vascular neurologic variables and the prevalence of MCI in patients with ARHL. The risk factors for MCI in patients with ARHL include cerebrovascular disease (OR = 1.41, 95% CI 1.17‒1.69, *p* < 0.00001), cardiovascular disease (OR = 1.29, 95% CI 1.07–1.55, *p* = 0.009), diabetes mellitus (OR = 1.28, 95% CI 1.20–1.35, *p* < 0.00001), head injury (OR = 1.22, 95% CI 1.13–1.33, *p* < 0.00001), alcohol consumption (OR = 1.28, 95% CI 1.14–1.43, *p* < 0.00001), and tobacco use (OR = 1.19, 95% CI 1.14–1.25).

### Psychological factors

**Figure 5:**
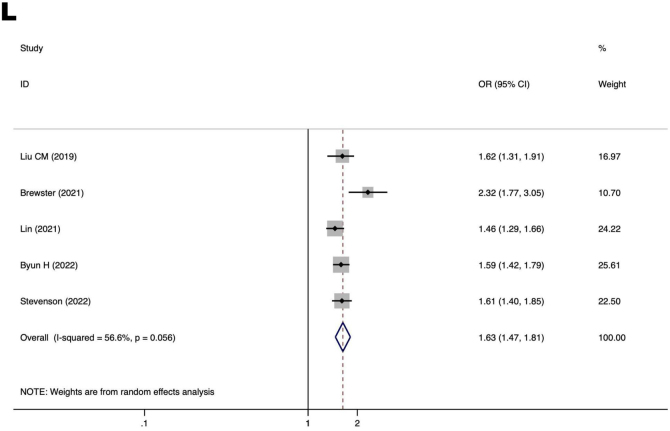
Forest plot of the relationship between psychological factors and MCI in ARHL patients. (L) Relationship between depression and MCI in patients with ARHL. OR, Odds Ratios; CI, Confidence Interval.

Fig. 5 displays a forest plot illustrating the correlation between psychological factors and the prevalence of MCI in individuals with ARHL. The risk factors for MCI in patients with ARHL was depression (OR = 1.63, 95% CI 1.47–1.81, *p* < 0.00001), indicating a highly significant result.

### Sensitivity analysis

The sensitivity analysis was conducted using a META analysis to assess the stability and credibility of the results. Two different effect models were used, and the combined effect size, OR, *p*, and 95% CI were compared before and after the analysis. The results showed that the results of the analysis were consistent, indicating good stability. The details of the sensitivity analysis can be found in Table 3.

**Table 3:** Sensitivity analysis of meta-analysis results.

Outcome indicator		Random effects model		Fixed effects model		Stability
		OR (95% CI)	*p*	OR (95% CI)	*p*	
Demographic characteristics	Age	1.63 (1.09, 2.43)	0.017	1.16 (1.15, 1.17)	0	Stabilize
	Amale	1.29 (1.14, 1.47)	0	1.31 (1.22, 1.41)	0	Stabilize
ARHL-related factors	Degree of hearing loss	1.35 (1.03, 1.75)	0.028	1.21 (1.13, 1.29)	0	Stabilize
	Course of hearing loss	1.31 (0.81, 2.14)	0.271	1.28 (0.93, 1.77)	0.127	Stabilize
	Not wearing hearing aids	1.51 (1.21, 1.88)	0	1.56 (1.37, 1.79)	0	Stabilize
Vascular neurological factor	Cerebrovascular disease	1.41 (1.17, 1.69)	0	1.57 (1.50, 1.63)	0	Stabilize
	Cardiovascular disease	1.29 (1.07, 1.55)	0	1.40 (1.34, 1.45)	0	Stabilize
	Diabetes mellitus	1.28 (1.20, 1.35)	0	1.28 (1.20, 1.35)	0	Stabilize
	Head injury	1.22 (1.13, 1.33)	0	1.22 (1.13, 1.33)	0	Stabilize
	Alcohol consumption	1.28 (1.12, 1.47)	0	1.28 (1.14, 1.43)	0	Stabilize
	Tobacco use	1.19 (1.13, 1.25)	0	1.19 (1.14, 1.25)	0	Stabilize
Psychological factor	Depression	1.63 (1.47, 1.81)	0	1.60 (1.49, 1.70)	0	Stabilize

## Discussion

ARHL has been demonstrated to be a distinct risk factor for cognitive decline, wherein MCI results in diminished quality of life for patients, increased burden of care for families, and substantial global expenditures. Defining risk variables is crucial for comprehending the connections between diseases, uncovering the underlying biopsychosocial medical models, and formulating prevention strategies and interventions. Extensive study has been conducted to assess the evidence supporting ARHL as a potential risk factor for the development of MCI. However, there is disagreement among studies on this matter. Thus, this study performed a Meta-analysis to examine the risk factors associated with MCI in individuals with ARHL. The analysis included 13 case-control studies and cohort studies of medium to high quality. The findings indicated that there were certain risk factors for MCI in patients with ARHL that could not be modified. These risk factors were characterized by demographic variables. On the other hand, there were other risk factors that could be modified, which included factors connected to ARHL, vascular neurological factors, and psychological factors.

The findings of this study indicate that the demographic parameters associated with risk factors for MCI in patients with ARHL include age and male gender. Several studies have demonstrated that the occurrence of MCI among individuals aged 60–84 years ranges from 6.7% to 25.2%,^5^, ^6^, ^7^ with a higher prevalence observed in older age groups. Additionally, a study conducted by Desjardins confirmed that the impact of hearing loss on cognitive functioning becomes more pronounced with advancing age,^27^ which aligns with the findings of this current study. The aging process is associated with the deterioration of memory neurons and decreased bodily function.^28^ It also leads to reduced social interaction and diminished auditory pathway activity in patients with ARHL. These changes in the brain’s structure put ARHL patients at a higher risk for MCI. Family members of individuals with ARHL should provide additional support, promote communication, enhance auditory pathway stimulation, and enhance cognitive function. The association between gender and the risk of MCI in patients with ARHL is now a topic of debate. In a study conducted by Fujita et al.,^29^ it was shown that the volume of the temporal lobe and hippocampus decreased in aged individuals. Furthermore, the rate of decline was greater in males compared to females. This suggests that male patients have a higher risk of MCI, which aligns with the findings of the current study. Nevertheless, a study by Nolan revealed that females can be considered a risk factor for MCI in patients with ARHL.^30^ This could be attributed to the potential protective influence of estrogen on hearing.^31^ However, it is important to note that after menopause, when estrogen levels decrease, women may experience a faster decline in hearing ability. This can lead to neuronal dysfunction and shrinkage of the auditory cortex, ultimately resulting in atrophy of the inner region of the medial temporal lobe.^32^ Several studies have suggested that there is a greater occurrence of MCI in women.^33^ This is likely due to the fact that women tend to have longer lifespans compared to males,^34^ and advancing age is a significant risk factor for the development of MCI. While there is a limited number of studies involving male patients with ARHL and MCI, it is advisable for future studies to consider gender as a significant risk factor.

The findings of this study indicated that the factors associated with ARHL that contribute to the development of MCI in ARHL patients are the severity and duration of hearing loss, as well as the absence of hearing aid usage. Yan et al.’s study found that the degree of hearing loss is a risk factor for MCI in patients with ARHL,^35^ which aligns with the findings of the current study. However, the specific mechanism by which the degree of hearing loss influences the development of MCI in ARHL patients remains a subject of debate.^36^ Various hypotheses, such as the “sensory deprivation” hypothesis,^37^ the “resource allocation” hypothesis,^38^ have been proposed to explain this relationship. Diao et al.’s study discovered that the course of hearing loss is a factor that increases the risk of cognitive impairment in individuals with ARHL.^39^ Additionally, Lin et al. observed that patients with ARHL exhibited cognitive structural changes in the right temporal lobe compared to six years ago,^40^ suggesting that the course of hearing loss has a substantial influence on the cognitive function of ARHL patients. There is evidence indicating that changes in the structure of the brain are related to cognitive function in people with ARHL. Additionally, the course of hearing loss appears to have an influence on cognitive function in these patients. The findings of this study contradict the findings of Diao et al.^39^ and Lin et al.,^40^ possibly because the study included a small number of studies and a large number of patients with other risk factors, which obscured the impact of the course of hearing loss on the disease. Additionally, a significant number of patients had a disease duration exceeding 6 years, and those with ARHL had already adapted to their living environment, resulting in a slower rate of cognitive decline. Therefore, it is recommended that future studies validate the categorization of the course of hearing loss. Several studies have shown that hearing aids have a positive impact on hearing ability as well as cognitive performance, depressive symptoms, and social functioning.^41^, ^42^, ^43^ Wearing hearing aids is the most straightforward and efficient method to address hearing loss.^25^ However, the utilization of hearing aids among individuals with ARHL is not widespread. This may be attributed to a lack of comprehension regarding the functionality of hearing aids, the financial burden,^44^ discomfort associated with wearing them, challenges in their usage, inadequate post-purchase support,^45^ and the absence of personalized features.^46^ It is advisable to enhance the public health system, intensify research on hearing aids, disseminate scientific knowledge on the subject, lower costs, enhance comfort, bolster after-sales service, and enhance personalized functionality in order to enhance the utilization of hearing aids in patients with ARHL.

The study findings indicate that vascular neurological factors contributing to the development of MCI in patients with ARHL encompass cardiovascular disease, diabetes, head injury, alcohol consumption, and tobacco use. Duncombe et al.,^47^ concurs with the findings of the current study, which demonstrated that cardiovascular disease results in inadequate cerebral perfusion. This leads to the buildup of β-amyloid in the brain, causing alterations in blood flow in the cochlea and vascular inflammatory damage,^48^ ultimately impacting hearing. Additionally, it results in brain disease,^47^ which in turn leads to a loss in cognitive function. The study found that people with diabetes mellitus experience continuous damage to blood vessels, nerves, tissues, and organs, as well as hearing and cognitive impairment, due to metabolic abnormalities.^49^ To prevent the occurrence of MCI in patients with ARHL who also have cardiovascular and cerebrovascular disorders and diabetes mellitus, it is advised to enhance the screening of cognitive function in their physical examination program. The findings of Azouvi et al.’s study align with the results of our study, indicating that head injury is a risk factor for the development of MCI in patients with ARHL.^50^ Furthermore, head injuries frequently lead to enduring neurological damage, with cognitive deficits being the predominant and persistent complication. Patients with ARHL have a diminished ability to avoid risks and are at a higher risk of head injury. It is advisable for ARHL patients to wear hearing aids as this can enhance their risk avoidance ability and decrease the likelihood of head injury. Additionally, wearing hearing aids can facilitate the restoration of the structure and function of the cerebral cortex, leading to improved cognitive function.^51^ Furthermore, it can mitigate the long-term effects of head injuries in ARHL patients and aid in their recovery from the disease. Adopting a healthy lifestyle can decrease the likelihood of MCI by affecting the cardiovascular system.^52^ Qian et al. demonstrated that consuming alcohol is a separate factor that increases the risk of hearing loss.^53^ Furthermore, Crespi et al.’s research revealed that alcohol consumption can result in abnormalities in the volume and density of gray matter, as well as white matter, neurodegenerative damage, and cognitive decline,^54^ which aligns with the findings of this study. A separate study demonstrated that those who consume alcohol in moderation exhibit superior cognitive abilities compared to both non-drinkers and individuals who drink alcohol daily.^55^ Conversely, excessive alcohol drinking escalates the likelihood of developing cancer, heart disease, other chronic ailments, and mental health disorders.^56^ The findings of this study indicate that smoking is a contributing factor to MCI in patients with ARHL. This aligns with the research conducted by Mons et al.^57^ and can be attributed to the detrimental effects of smoking on the brain’s gray matter density,^58^ cardiovascular system,^59^ and induction of oxidative stress,^60^ among other factors. Over time, it is advisable for individuals with ARHL to adopt a healthful way of life and manage their consumption of tobacco and alcohol. This not only diminishes the likelihood of enduring chronic ailments and mental health issues, but also enhances hearing impairment and cognitive abilities.

The study revealed that depression is the primary psychological factor contributing to MCI in patients with ARHL. This finding aligns with the results of Morimoto et al.,^61^ which demonstrated that depression causes structural alterations in the cerebral cortex and elevates inflammatory markers. Consequently, these changes lead to cognitive decline and heighten the likelihood of MCI in ARHL patients. Additionally, the study found that hearing loss amplifies the risk of depression,^40^ thereby establishing a detrimental cycle. ARHL patients should engage in tertiary prevention methods to manage hearing decline and decrease depression levels. Additionally, working with psychiatric healthcare professionals can help ARHL patients recognize that hearing loss is treatable and not an inevitable consequence of aging. This can help establish accurate knowledge about hearing loss treatment, including addressing concerns about discomfort from wearing hearing aids. These efforts can contribute to reducing the risk of MCI in ARHL patients.

This study also has some limitations. First, limitations in the quantity and quality of literature may have led to biased data. Second, the small sample size in some of the studies may have affected the reliability of the results. In addition, the diversity of the studies involved, including differences in geographic and demographic characteristics, may have affected the generalization of the results.

## Conclusion

This meta-analysis aimed to thoroughly investigate and analyze the risk factors associated with MCI in ARHL patients. Our findings showed that demographic characteristics, ARHL-related factors, vascular neurologic factors, and psychological factors were significantly correlated with MCI in patients with ARHL. These findings have significant clinical implications for the prevention and treatment of MCI in patients with ARHL.

## Authors’ contributions

Data collection: Wenjuan Wang and Chenxingzi Wu.

Data analysis and draft writing: Chenxingzi Wu.

Technical support, study design and supervision: Ruilin Li, Yuhong Su, Huiling Lv, Shuhong Qin and Zhanhang Zheng.

## Funding

This study was supported by the Doctoral Research Initiation Fund Project of Guangxi University of Chinese Medicine (Grant No. 2023BS056).

## Conflicts of interest

The authors declare no conflicts of interest.
